# Transcriptome Analysis in Hippocampus of Rats Prenatally Exposed to Valproic Acid and Effects of Intranasal Treatment of Oxytocin

**DOI:** 10.3389/fpsyt.2022.859198

**Published:** 2022-03-30

**Authors:** Kazuya Matsuo, Yasuharu Shinoda, Nona Abolhassani, Yusaku Nakabeppu, Kohji Fukunaga

**Affiliations:** ^1^Department of Pharmacology, Graduate School of Pharmaceutical Sciences, Tohoku University, Sendai, Japan; ^2^Division of Neurofunctional Genomics, Department of Immunobiology and Neuroscience, Medical Institute of Bioregulation, Kyushu University, Fukuoka, Japan

**Keywords:** autism spectrum disorders, hippocampus, oxytocin, transcriptome analysis, valproic acid

## Abstract

Autism spectrum disorder (ASD) is a heterogeneous disorder characterized by repetitive behaviors and social impairments, often accompanied by learning disabilities. It has been documented that the neuropeptide oxytocin (OXT) ameliorates core symptoms in patients with ASD. We recently reported that chronic administration of intranasal OXT reversed social and learning impairments in prenatally valproic acid (VPA)-exposed rats. However, the underlying molecular mechanisms remain unclear. Here, we explored molecular alterations in the hippocampus of rats and the effects of chronic administration of intranasal OXT (12 μg/kg/d). Microarray analyses revealed that prenatal VPA exposure altered gene expression, a part of which is suggested as a candidate in ASD and is involved in key features including memory, developmental processes, and epilepsy. OXT partly improved the expression of these genes, which were predicted to interact with those involved in social behaviors and hippocampal-dependent memory. Collectively, the present study documented molecular profiling in the hippocampus related to ASD and improvement by chronic treatment with OXT.

## Introduction

Autism spectrum disorder (ASD) is a neurodevelopmental disorder characterized by social deficits and repetitive behaviors (1). ASD involves heterogeneous and complex causal factors, both genetically and environmentally. While it has been documented that various copy numbers or single nucleotide variations are associated with ASD, multiple environmental factors such as maternal infection, exposure to drugs or toxicants, and immune dysregulation have also been implicated (2). Such diverse causes, their interactions, and the resultant complex symptoms make it difficult to focus on particular targets and therapeutic approaches in ASD.

The neuropeptide oxytocin (OXT) facilitates socio-communicative behaviors in mammals (3). OXT or OXT receptor deficiency impairs multiple social behaviors (4, 5). In addition, plasma levels of OXT are significantly lower in children with autism than in their normal counterparts (6, 7). Clinical studies have documented that the intranasal administration of OXT improves socio-emotional impairments in patients with ASD (8, 9). Thus, OXT could be considered a suitable candidate for treating the core symptoms in ASD.

Maternal use of the anti-epileptic drug valproic acid (VPA) during pregnancy is suggested to increase the risk of teratogenicity and ASD onset in offspring (10, 11). These features are reproducible in animals; they represent defects in the limbs and tail and ASD-like social deficits (12, 13). In addition, transcriptome analyses have identified some key molecular pathways for ASD in the amygdala and prefrontal cortex of the model (12, 14, 15); alteration in signaling of protein kinase A and Rho GTPases in the amygdala and calcium signaling in the prefrontal cortex. These pathways are related to synaptic plasticity, a pathological hallmark in ASD (16–19), suggesting that molecular alterations in these emotion-related regions are involved in the pathogenesis of ASD. Especially, GTPases signaling defects are well documented in neurodevelopmental disorders including ASD (20); Rho GTPase Cdc42, a regulator of neurite outgrowth, is reduced in autistic patients (21). Prenatal VPA exposure decreases mRNA levels of Rho GTPase-activating protein p250GAP (22). In addition, p21-activated protein kinase exchange factor, a Rho GTPase regulatory protein, interacts with SH3 and multiple ankyrin repeat domains (SHANK) proteins in spines to regulate postsynaptic structure (23). Since mutations in SHANK gene have been associated with neurodevelopmental and neuropsychiatric disorders, including ASD (24), it is noteworthy that recent findings show that OXT restores neurite abnormalities in hippocampal cultures with SHANK3 deficiency through amelioration of Rho GTPase levels (25). Thus, prenatally VPA-exposed animals are a valid ASD model in terms of behavioral phenotypes and epigenetic modulation of gene expression as an environmental factor.

In our previous studies, we had reported that prenatal VPA exposure impairs learning and long-term potentiation (LTP) in the hippocampus of rats and that chronic administration of intranasal OXT ameliorates learning disabilities (26, 27). In line with our observations, intranasal administration of OXT blocked the learning disability in prenatally VPA-exposed or restraint stress-exposed animal models (28–30). In contrast, Hara et al. demonstrated that a single dose of OXT was effective for social impairment only for a short period and not for memory impairment (31). These reports suggest that the mechanisms of action of chronic OXT would involve molecular alterations in addition to the acute activation of oxytocinergic signaling. However, it remains unclear which molecular pathways are affected by chronic administration of OXT. Furthermore, molecular profiling in the dorsal hippocampus, a critical region for learning and memory, and partly implicated for social behaviors (32, 33) has not been investigated in a prenatally VPA-exposed model.

In the present study, we explored transcriptome profiling in the hippocampus of prenatally VPA-exposed rats. It was seen that prenatal VPA exposure altered the expression levels of genes involved in multiple behaviors and developmental behaviors, some of which were documented as candidate genes in ASD. We also demonstrated that the chronic administration of intranasal OXT partly ameliorated these alterations.

## Materials and Methods

### Animals

Animal studies conformed to the Regulations for Animal Experiments and Related Activities at Tohoku University and were approved by the Committee on Animal Experiments at Tohoku University (approval number: 2020PhA-007). Every effort was made to use minimum the number of rats and minimize their discomfort. Animals were bred in a conventional environment (temperature, 21–23°C; humidity, 50–60%; 12-h light-dark cycle) with free access to normal chow and water. Three pregnant Sprague-Dawley rats (Japan SLC, Shizuoka, Japan) received a single administration of oral VPA (600 mg/kg; Sigma-Aldrich, St. Louis, MO, United States) on day 12.5 as described previously (27). Two control rats received water in the same way. Rats gave birth from 7 to 14 pups per litter, of which 3–8 were males (sex ratio of male to female was about 1:1.1). Sizes and body weights of pups were not affected by VPA treatment, as reported by a previous study (26). Only male pups were included in this study because of the higher incidence of ASD in males than in females and even in prenatally VPA-exposed models (34).

### Oxytocin Treatment

On day 21 of birth, the rats were randomly divided and subsequently received vehicle or OXT administration: 3 pups from 2 control rats and 6 pups from 3 VPA-treated rats, with the latter further divided into 3 vehicle- or OXT-treated groups of 3 pups each. Male pups received intranasal OXT (Peptide Institute, Osaka, Japan) dissolved in saline at a dose of 12 μg/kg/d, which is in a range of that promoting social behaviors in rodents (35–37), using a pipette tip on postnatal day 21–55. The liquid volume of OXT solution was changed in the range of 2–10 μL according to the growth of rats. The dose and period of treatment were the same as those in a previous study where OXT attenuated autistic behaviors in prenatally VPA-exposed rats (27).

### Identification of Differentially Expressed Genes in Microarray Analysis

Total RNA was extracted from the dorsal hippocampus on postnatal day 56. As with most transcriptome analyses, the number of samples per condition is 2–5 for the analyses using VPA-treated ASD models (12, 14, 15). Therefore, we decided that three samples per condition would be sufficient for this study. Expression profiles were determined using Rat Gene 2.0 ST array systems (Affymetrix, Santa Clara, CA, United States) and analyzed using Transcriptome Analysis Console software (version 4.0; RRID:SCR_018718; Affymetrix). Differentially expressed genes (DEGs) were evaluated as follows: (1) *P* < 0.05, in a one-way analysis of variance computed by limma (38), (2) more than 1.5 fold-change (both increase and decrease) between control and vehicle-treated VPA groups. (3) Expression levels (in log_2_ scale) more than 6.6 at least in one group. Among these, significantly different (improved) values between the vehicle-treated VPA and OXT-treated VPA groups were further investigated. Expression levels were normalized to those of the control group. Microarray data were deposited in the GEO database (accession number: GSE196500).

### Gene Ontology Enrichment Analysis

Gene ontology (GO) enrichment analysis was performed using Metascape (RRID:SCR_016620^1^) (39). To obtain the most comprehensive data, gene identifiers were converted from *Rattus norvegicus* to *Homo sapiens* orthologs. GO terms classified in biological processes were collected under the following conditions: a minimum count of 3, *P* < 0.01, and an enrichment factor (the ratio between the observed counts and the counts expected by chance) > 1.5. Cytoscape App (RRID:SCR_003032) was used to create and plot the enrichment network (40).

### Protein-Protein Interaction Enrichment Analysis

To investigate protein networks consisting of proteins that form physical interactions each other in DEGs, protein-protein interaction analysis was performed using databases including BioGrid (RRID:SCR_007393) (41), InWeb_IM (42), and OmniPath (43) in Metascape. The molecular complex detection (MCODE) algorithm (RRID:SCR_015828) (44) was applied to identify tightly connected modules in each network. MCODE analysis was performed with default settings; detection when there are at least three genes in a network.

### Gene-Disease Association and Regulatory Interaction Analysis

To reveal the involvement of DEGs in diseases and their transcriptional regulation, DisGeNET (RRID:SCR_006178) (45) and TRRUST (46) were performed in Metascape. The algorithm was the same as that of the GO enrichment analysis discussed above.

### Overlapping With Autism Spectrum Disorder Risk Genes and Predictive Analysis of Interaction

Two databases were used to explore the overlap between DEGs in the VPA model and candidate genes in ASD patients: SFARI (RRID:SCR_004261^2^) and Krishnan’s datasets Genome-wide predictions of autism-associated genes^3^ (47). Gene lists in SFARI were scored as follows: S (syndromic), 1 (high confidence), 2 (strong candidate), and 3 (suggestive evidence). In Krishnan’s dataset, gene list associated with ASD are separated between brain regions and developmental periods, and the hippocampus in middle-late childhood (ID: HIP. 11) was selected to be appropriate for comparison with the results of this study. Additionally, to predict the interactive networks between DEGs improved by OXT and ASD candidate genes, Krishnan’s dataset was used.

## Results

### Gene Expression Profiles in the Hippocampus of Prenatally Valproic Acid-Exposed Rats

**FIGURE 1:**
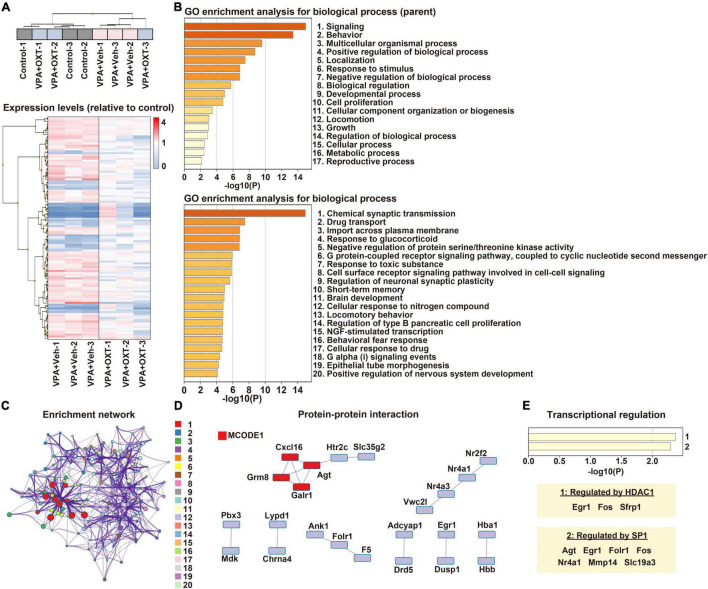
Transcriptome profiling in the hippocampus of prenatally VPA exposed rats. **(A)** Hierarchical clustering of DEGs between groups. Expression levels were normalized to those of the control. **(B)** Biological processes of DEGs in GO enrichment analysis. Top, parent; Down, child annotations, respectively. **(C)** Enrichment networks between clusters of biological processes. Shown numbers are the same with those of child annotations in panel **(B)**. **(D)** Protein-protein interactive networks between DEGs. Shown by MCODE1 represents a strongly connected network. **(E)** Analysis of transcriptional regulation of DEGs. HDAC1 and SP1 are detected as significant regulators. VPA, valproic acid; DEG, differentially expressed gene; GO, gene ontology; MCODE1, molecular complex detector; HDAC, histone deacetylase; SP1, specificity protein 1.

Gene expression profiles of the hippocampus were compared between the control, vehicle-treated VPA, and OXT-treated VPA groups (*n* = 3 per group). Hierarchical clustering tended to separate the vehicle-treated VPA group from the others, except for one sample from the OXT-treated VPA group (Figure 1A). Microarray analysis revealed that 377 genes differed significantly by more than 1.5-fold between the groups (Figure 1A). Among these, 174 genes were considered DEGs (see section “Materials and Methods”). We then analyzed the biological processes of DEGs between the control and vehicle-treated VPA groups. GO enrichment analysis revealed that DEGs were roughly associated with multiple functions, including signaling, behavior, and developmental processes (Figure 1B). An in-depth analysis further indicated that DEGs are involved in chemical synaptic transmission, short-term memory, brain development, as well as nervous system development (Figure 1B). These results suggest that prenatal VPA exposure affects multiple genes in the hippocampus that are involved in synaptic function, learning and memory, and neurodevelopment, all of which are the key features of ASD. In particular, the enrichment network analysis also indicated that chemical synaptic transmission (#1) serves as a hub function in these biological processes (Figure 1C). Protein-protein interaction analysis further predicted that several interactive networks are formed among the DEGs (Figure 1D). Interestingly, histone deacetylase 1 (HDAC1) and specificity protein 1 (SP1), both of which are regulated by VPA (48, 49), were identified as factors for the transcriptional regulation of DEGs (Figure 1E).

### Autism Spectrum Disorder-Associated Molecular Changes in the Hippocampus by Prenatal Valproic Acid Exposure

**FIGURE 2:**
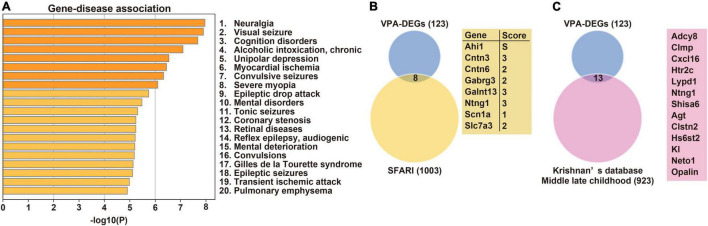
Disease-associated profiling and overlap with ASD candidate genes. **(A)** Association between DEGs and human diseases. Venn’s diagrams depict overlap between DEGs and candidate genes for ASD registered in SFARI **(B)** and Krishnan’s database **(C)**, respectively. ASD, Autism spectrum disorder; DEG, differentially expressed gene.

We next addressed which human diseases are associated with DEGs. Notably, DEGs are known to be enriched in ASD-associated [mental disorders, Gilles de la Tourette syndrome (50), and various types of epilepsy] and learning disability-associated (cognition disorders and mental deterioration) diseases (Figure 2A). To further reveal the relationship with human ASD, the overlap between DEGs and ASD candidate genes was investigated using two databases. Eight DEGs were included in the SFARI database, and *Ahi1* was indicated as a cause of syndromic ASD (Figure 2B). In addition, 13 DEGs overlapped with gene sets in the hippocampus of ASD in middle-late childhood in Krishnan’s database (Figure 2C). Collectively, these results suggest that molecular profiles in the hippocampus could underlie autistic behaviors, including learning disabilities seen in prenatally VPA-exposed rats.

### Effects of Chronic Administration of Intranasal Oxytocin on Gene Expression in the Hippocampus of Prenatally Valproic Acid-Exposed Rats

**FIGURE 3:**
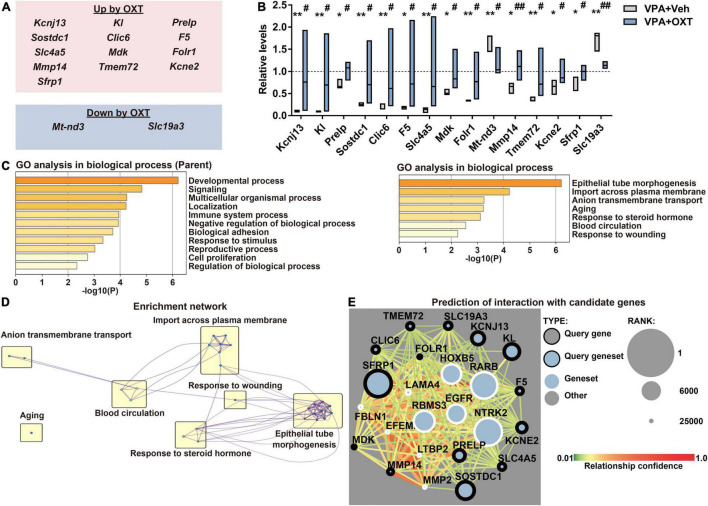
Effect of chronic treatment of intranasal OXT on prenatal VPA exposure-induced molecular changes. **(A)** Summary of (top) upregulated and (bottom) downregulated DEGs by OXT (12 μg/kg/day). **(B)** Quantification of expression levels of genes improved by OXT. The levels were normalized to those of the control. The top and bottom of the bar depict the highest and the lowest of values, respectively. The line within the bar shows the median (*n* = 3 per group). *P < 0.05 and **P < 0.01 vs. control; ^#^*P* < 0.05 and ^##^*P* < 0.01 vs. vehicle-treated VPA. **(C)** Biological processes of DEGs in GO enrichment analysis. Left, parent; Right, child annotations, respectively. **(D)** Enrichment networks between clusters of biological processes with child annotations. **(E)** Predictive interaction between DEGs improved by OXT and ASD candidates in Krishnan’s database. Shown are top 10 of candidates connecting strongly with our gene sets. DEG, differentially expressed gene; OXT, oxytocin; GO, gene ontology.

To explore the molecular mechanisms by which chronic OXT treatment improves the prenatal VPA exposure induced autistic behaviors, significantly improved populations by OXT among DEGs were extracted (Figure 3A). Chronic administration of intranasal OXT (12 μg/kg/d) significantly upregulated 13 genes and downregulated two genes among the DEGs (Figure 3B). GO enrichment analysis revealed that these improved genes belonged to partly similar processes with DEGs (Figure 1B), including the developmental process, signaling, and import across the plasma membrane, and different genes such as those involved in epithelial tube morphogenesis and response to steroid hormone (Figure 3C). These GO genes formed networks with each other except for aging, and epithelial tube morphogenesis, and thus would serve as a hub annotation (Figure 3D). Finally, interactive networks were predicted between ASD candidates and DEGs improved by OXT using Krishnan’s database (except for *Mt-nd3*, which were not registered in the database). The predicted top 10 genes that interacted with 14 of the DEGs improved by OXT are shown in Figure 3E. Of these, three genes were reported to be involved in social behaviors, learning, and memory (Table 1). Chronic administration of intranasal OXT has the potential to partly regulate the molecular pathways in the hippocampus involved in ASD-like behaviors induced by prenatal VPA exposure.

**TABLE 1:** Relationship of the predicted genes to hippocampal function and ASD-like behaviors.

Genes	Function in the hippocampus and ASD phenotypes	Sources
*Egfr*	Enhancement of LTP through recruitment of NMDA receptor GluN2B subunit by EGFR activation	(68)
*Rarb*	Impairments of LTP, AMPA receptor-mediated synaptic transmission, spatial memory, and social recognition by reduction in RARβ levels	(74)
*Ntrk2*	Impairments of hippocampal LTP and learning in TrkB knockout mice; Reduction of TrkB levels and LTP in the hippocampus and impaired learning and sociability in an inbred ASD model	(69, 70)

*AMPA, α-amino-3-hydroxy-5-methyl-4-isoxazolepropionic acid; EGFR, epidermal growth factor receptor; NMDA, N-methyl-D-aspartate; RARβ, retinoic acid receptor β; TrkB, tropomyosin receptor kinase B.*

## Discussion

Valproic acid is an inhibitor of HDACs that epigenetically modulates gene expression (48). It has been suggested that VPA-induced teratogenicity results from HDAC inhibition. The effects of HDAC inhibitors on fetal teratogenicity are suppressed by their analogs with lower potency against HDACs (51, 52). Interestingly, it has been reported that the prenatal VPA exposure-induced social deficits were ameliorated by chronic administration of HDAC inhibitors at postnatal days (13), supporting the critical role of epigenetic modulation by HDAC inhibition for both onset and amelioration of ASD. In the present study, HDAC1 and specificity protein 1 were implicated as transcriptional regulators of DEGs, suggesting the relevance of the hippocampus in ASD-like molecular profiling and behaviors induced by prenatal VPA exposure.

The dorsal and ventral hippocampus primarily function in cognition and emotion, respectively; inhibition of protein synthesis in the dorsal hippocampus decreases fear memory consolidation (53). The dorsal hippocampus lesion also impairs spatial memory (54). Optogenetics experiments suggested that activity in the ventral hippocampus is required for social memory recall in mice (55). In terms of emotional behaviors, in contrast, the dorsal and ventral parts are reported to oppositely regulate anxiety in rodents; muscimol infusion into the dorsal hippocampus provokes anxiety, while into the ventral part has anxiolytic effects (56). In addition, dorsal-ventral neural circuits in the hippocampus contribute to social memory (57). These reports suggest that the dorsal hippocampus is also involved in emotion, although the degree of contribution is likely to be less than in the ventral part. It is noteworthy that hippocampal volume is altered in ASD patients compared to typically developed individuals, although there is a discrepancy whether the volume increases or decreases (58, 59). Functional magnetic resonance imaging showed that neural connectivity in the anterior hippocampus (ventral part in rodents) was reduced in ASD patients (60). However, there are few reports on the anatomical and functional findings of the dorsal hippocampus in ASD patients.

In this study, 174 genes were identified as DEGs in the hippocampus of prenatally VPA-exposed rats. Among these, only 32 genes were also identified as DEGs in the amygdala or prefrontal cortex of prenatally VPA-exposed models (12, 14, 15), suggesting a distinct pattern of molecular changes between the brain regions in models such as ASD patients (47). According to the gene-disease analysis, prenatal VPA exposure affects a subset of genes in the hippocampus involved in both ASD- and learning disability-associated diseases (Figure 2A). Using transcriptome profiling, our study thus demonstrated that the hippocampus is a key region in terms of its contribution to ASD phenotypes, including learning disabilities.

We identified some key molecular changes induced by prenatal VPA exposure and upregulated by OXT. Secreted frizzled-related protein 1 is an endogenous inhibitor of Wnt signaling (61), which gets activated in both the brains of ASD patients and the hippocampus of prenatally VPA-exposed rats (62, 63). Interactive networks also revealed a relatively higher connection between *Sfrp1* and *Rarb* (Figure 3E). Transcription of cluster of differentiation 38, which is critical for OXT release (64), is regulated by RARs (65). The severity of ASD has been reported to negatively correlate with serum levels of vitamin A (66). Notably, Lai et al. reported that maternal deficiency of vitamin A in rats induced ASD-like behaviors and decreased the expression levels of cluster of differentiation 38 and retinoic acid receptor β in the hypothalamus and OXT in the serum of the offspring, all of which were rescued by maternal supplementation of vitamin A (67). These reports suggest that the molecular pathways involved in Wnt signaling, including secreted frizzled-related protein and retinoic acid receptor β, play a critical role in ASD pathology and improve following chronic OXT treatment. Epidermal growth factor receptor and tropomyosin receptor kinase B were also predicted to interact with DEGs improved by OXT (Figure 3E and Table 1), and these genes are reported to be involved in social behaviors, learning, and hippocampal LTP (68–70). Oxytocinergic signaling activates epidermal growth factor receptor to promote LTP maintenance (71). Both *Ntrk* deletion, specifically in oxytocinergic neurons and *Bdnf* deletion, impair maternal behaviors against offspring in mice (72). These reports support that OXT is involved in the molecular pathways underlying not only social behaviors but also hippocampus-dependent learning and memory. To the best of our knowledge, this is the first report to evaluate effects of OXT on molecular alterations in the dorsal hippocampus of an animal model of ASD. However, a limitation of this study is that it did not evaluate the effects of OXT on molecular alterations in the amygdala and prefrontal cortex, regions related to emotion and well-investigated in ASD. As mentioned above, it is likely that the pattern of molecular alterations is distinct between brain regions in ASD. In order to further confirm the efficacy of OXT for ASD, the effects on molecular alterations in these regions should be investigated in the future.

In this study, chronic administration of OXT was conducted at adolescence just after weaning in order to avoid the risk of parental abandonment. Interestingly, maternal administration of OXT is implicated to suppress postnatal pathogenesis in animal models of ASD through enhancing excitatory/inhibitory switching of γ-aminobutyric acid during development (73). This suggests the possibility of maternal administration of OXT may reduce the risk of postnatal development of ASD, even when epilepsy patients are medicated with anticonvulsants including VPA during pregnancy. Further study is needed in the future to investigate the possibility of maternal OXT medication for restoration of postnatal ASD development and molecular alterations.

In summary, the present study demonstrated that prenatal VPA exposure affects the molecular pathways involved in ASD-like phenotypes in the hippocampus. Chronic administration of intranasal OXT partly ameliorated these alterations, which would underlie the improvement of social and learning disabilities seen in the prenatally VPA-exposed ASD model.

## Data Availability Statement

The datasets presented in this study can be found in online repositories. The names of the repository/repositories and accession number(s) can be found below: https://www.ncbi.nlm.nih.gov/geo/query/acc.cgi?acc=GSE196500.

## Ethics Statement

The animal study was reviewed and approved by the Committee on Animal Experiments at Tohoku University.

## Author Contributions

KM and YS performed the experiments. NA and YN verified the methodology and provided the materials and apparatus. KM and KF wrote the manuscript. KF designed the study. All authors contributed to the article and approved the submitted version.

## Conflict of Interest

The authors declare that the research was conducted in the absence of any commercial or financial relationships that could be construed as a potential conflict of interest.

## Publisher’s Note

All claims expressed in this article are solely those of the authors and do not necessarily represent those of their affiliated organizations, or those of the publisher, the editors and the reviewers. Any product that may be evaluated in this article, or claim that may be made by its manufacturer, is not guaranteed or endorsed by the publisher.

## Footnotes

1
http://metascape.org

2
https://gene.sfari.org/

3
http://asd.princeton.edu

## Abbreviations

ASD: autism spectrum disorder
DEG: differentially expressed gene
GO: gene ontology
HDAC: histone deacetylase
LTP: long-term potentiation
MCODE: molecular complex detection
OXT: oxytocin
SHANK: SH3 and multiple ankyrin repeat domains
VPA: valproic acid.
